# Trained immunity and immune priming in plants and invertebrates

**DOI:** 10.7554/eLife.106597

**Published:** 2025-12-05

**Authors:** Joachim Kurtz, Raul Andino, Diana Boraschi, Jorge Contreras-Garduño, Aardra Kachroo, Imroze Khan, Humberto Lanz Mendoza, Krishnendu Mukherjee, Robert Peuß, Jurriaan Ton

**Affiliations:** 1 Institute for Evolution and Biodiversity, University of Münster Münster Germany; 2 Department of Microbiology and Immunology, University of California, San Francisco San Francisco United States; 3 Shenzhen University of Advanced Technology Shenzhen China; 4 Shenzhen Institutes of Advanced Technology, Chinese Academy of Sciences Shenzhen China; 5 National Research Council Naples Italy; 6 Stazione Zoologica Anton Dohrn Naples Italy; 7 Escuela Nacional de Estudios Superiores, Universidad Nacional Autónoma de Mexico Morelia Mexico; 8 Department of Plant Pathology, University of Kentucky Lexington United States; 9 Trivedi School of Biosciences, Ashoka University Sonepat India; 10 Instituto Nacional de Salud Pública. Centro de Investigaciones sobre Enfermedades Infecciosas Cuernavaca Mexico; 11 Institute of Hygiene, University of Münster Münster Germany; 12 Institute for Integrative Cell Biology and Physiology, University of Münster Münster Germany; 13 School of Biosciences University of Sheffield Sheffield United Kingdom; Münster University Hospital Germany; National Institute of Immunology India

**Keywords:** immune memory, immune priming, trained immunity, invertebrates, plants, innate immunity

## Abstract

Immune memory has long been thought to be restricted to the adaptive immune system of vertebrates. However, several lines of evidence have changed our understanding of immune memory and have shattered the strict separation between innate and adaptive immunity. In vertebrates, a form of innate immunity that is called ‘trained immunity’ has been intensively studied for over a decade. For more than two decades, studies in plants and an increasing number of invertebrate taxa have clearly demonstrated that these organisms also possess immune memory, despite the absence of an adaptive immune system. These phenomena are mostly known as ‘immune priming’. The mechanistic underpinnings of immune priming vary across taxa and may or may not partially include the epigenetic and metabolic mechanisms involved in trained immunity. Here, we offer an evolutionary perspective on immune priming, uniquely integrating key aspects across plants and invertebrates for the first time. As a basis, we provide a conceptual clarification regarding the terms trained immunity and immune priming and give a brief overview of these phenomena across taxa. We then analyze which processes of immune priming share potentially evolutionary conserved epigenetic and metabolic processes with trained immunity and explore signaling processes involved in immune priming. We discuss the aspect of specificity as one of the key defining criteria for immune memory and incorporate the potential role of soil and gut microbiota for acquiring innate immune memory in plants and invertebrates. Finally, we argue that immune priming has enormous potential for application beyond the medical field when involving the protection against parasites and pathogens in agriculture and aquaculture.

## Introduction

Since the inception of modern immunology, adaptive immunity and immune memory have been classically viewed as a protective response against invading pathogens that is exclusive to vertebrates (Murphy et al., 2022). Adaptive immunity is based on mechanisms of somatic recombination for the generation of diversity that appeared in early vertebrates, and for generating long-lived memory cells specific for the infectious challenges (Boehm, 2025; Cooper, 2018). By contrast, invertebrates and plants were considered to possess a relatively straightforward and non-specific immune system, because they lack specialized immune memory cells, such as B and T cells, and analogues of highly specific effector molecules such as antibodies. The paradigm was that vertebrates possess two immune systems, the newer specific adaptive immunity, able to generate memory, and the evolutionarily older non-specific ‘innate’ immunity similar to that of invertebrates, unable to generate memory. As a result, the possibility that invertebrate immunity may have memory has generally been overlooked in immunology textbooks. Although earlier literature from the 1960 s-1980s, including studies on crustaceans such as crayfish (McKay et al., 1969), terrestrial annelids like earthworms (Cooper and Roch, 1986) and insects such as *Drosophila melanogaster* (Boman et al., 1972) and American cockroaches (Hartman and Karp, 1989) indicated the potential for immune memory to be part of the invertebrate immune response, it was not until the first decade of the 20th century that this concept gained wider attention. Seminal work by Kurtz and Franz, 2003 demonstrated highly specific, immune memory-like responses in the copepod *Macrocyclops albidus* against its natural parasite tapeworm *Schistocephalus solidus*. These results initially appeared counterintuitive due to the apparent absence of traditionally known immune cells that could generate memory in these animals. However, soon after, numerous studies on other invertebrates showed that previous sublethal exposure to a pathogen could indeed offer protection against re-infection within or across life stages or even in offspring both in the laboratory and in wild populations (reviewed in Milutinović and Kurtz, 2016; Prakash and Khan, 2022; Rutkowski et al., 2023). For example, the phenomenon was described in dipterans (Gomes et al., 2022; Pham et al., 2007) coleopterans (Roth et al., 2009), lepidopterans (Fallon et al., 2011), hymenopterans (Sadd and Schmid-Hempel, 2006), arachnids (Gálvez et al., 2020), molluscs (Arfatahery et al., 2024), nematodes (Yan et al., 2020), and even cnidarians (Brown and Rodriguez-Lanetty, 2015), suggesting the general importance of innate immune memory across many invertebrate species. Immune priming is thus an integral immune feature of invertebrates.

Abundant evidence shows that plants can also bolster their resistance to infections through priming-like responses that enable them to respond more quickly and effectively against future infections. Such responses do not rely on specialized immune cells but can be initiated in all plant cells through carefully orchestrated phytohormone-, metabolite-, protein-, and RNA-based signaling, providing durable protection that can be active at spatially distant sites through systemically transported signals. The finding that plants can exhibit increased immunity due to prior exposure to a pathogen was recorded as early as the 1930s (Chester, 1933). A subsequent pioneering study showed that challenge inoculation with Tobacco mosaic virus (TMV) resulted in enhanced resistance to subsequent infections by TMV as well as four other viruses in tobacco (Ross, 1961). A series of systematic studies followed to show that this type of immune memory could be activated in all plant parts and was effective against a broad spectrum of pathogen types (Bergstrom, 1982). Although systemic immune memory has been most studied in non-woody angiosperms and gymnosperms, bryophytes too exhibit this type of immunity, suggesting that this mechanism evolved before the divergence of vascular and non-vascular plants and is at least >515–494 million years old (Bonello, Gordon, Storer, 2001; Krokene et al., 1999; Ryals et al., 1996; Winter et al., 2014).

The description of protective priming in the various organisms warrants a clear definition of immune memory and how to characterize it. For instance, in vertebrates, immune memory is complex, with the highly specific adaptive memory relying on memory B and T cells (Murphy et al., 2022), while immune priming in invertebrates and plants is based on different mechanisms (Cooper and Ton, 2022; Milutinović and Kurtz, 2016), although in general the distinction in their functional outcomes is not clear-cut. It should be stressed that adaptive immunity is present in vertebrates, which represent less than 5% of all animal species on Earth, and that, in vertebrates, adaptive immunity is engaged in combating only a low proportion of incoming pathogens, while all the others can be successfully eliminated by innate immunity (Gourbal et al., 2018; Murphy et al., 2022). We should therefore consider immune memory in invertebrates and plants (and the innate immune memory in vertebrates) as a major defensive mechanism maintained in evolution, while adaptive immune memory of vertebrates is only a recent refinement of protective memory for specifically tackling some highly adapted pathogens (able to circumvent innate memory). In mammals, innate immune memory is rapidly becoming a central topic in immunology (Netea et al., 2020) for its implications as a possible factor in disease development and as a tool in preventive strategies. Experimental evidence indicates that an initial vaccination or infection by bacteria, fungi, or helminth parasites can prime mammalian innate immune cells (such as monocytes, macrophages, or innate lymphoid cells) through epigenetic modifications and metabolic changes, to generate an immune memory profile that enables them to tackle future infections independently of the adaptive immune response (Ochando et al., 2023). For instance, in immunodeficient mice that lack T and B lymphocytes, vaccination with live BCG (the human vaccine for *Mycobacterium tuberculosis*) can provide nonspecific protection against *Candida albicans* infection (Kleinnijenhuis et al., 2012). Also, injection of fungal cell wall components (e.g. β-glucan) can attain enhanced protection in mice against acute *Staphylococcus aureus* (Di Luzio and Williams, 1978) or *M. tuberculosis* infection (Moorlag et al., 2020). It is thus clear that immune memory is a primary protective mechanism in all multicellular life forms, while adaptive memory of vertebrates is a relatively recent development of it. Various life forms facing the selection pressure of recurrent infections should evolve a robust immune memory against pathogens (Lui, 2000), using diverse mechanisms, which warrants a deeper evolutionary and mechanistic exploration.

## Conceptual clarifications

The terms ‘trained immunity’ and ‘immune priming’ are currently used interchangeably without a clear understanding of the commonalities and differences between these phenomena (e.g. Kulkarni et al., 2021; Montagnani et al., 2024). Likewise, the term innate immune memory has been used based on different, broad or narrow, definitions of memory. This shows that the field may still benefit from an attempt for conceptual clarification, even though a number of in-depth articles have already addressed the concepts of innate immunity (Pradeu et al., 2024), trained immunity (Divangahi et al., 2021; Netea et al., 2020), immune memory (Natoli and Ostuni, 2019; Pradeu and Du Pasquier, 2018) and memory within innate immunity (Boraschi and Italiani, 2018; Boraschi et al., 2024; Kurtz, 2004; Kurtz, 2005; Lanz-Mendoza and Contreras-Garduño, 2022; Lanz-Mendoza et al., 2024).

Immune memory has been broadly defined as the ability to recognize and respond more rapidly and effectively to a parasite or pathogen that was encountered before. Such protection to hosts during subsequent encounters usually decreases the likelihood of reinfection and enhances host survival (Kurtz, 2005; Murphy et al., 2022). Immune memory thus provides a learned record of past infections. As simple as this definition may seem, it reveals several intricacies upon closer inspection.

First, we would like to point out that the term memory refers to a multidimensional and gradual phenomenon that cannot be reduced to a simple dichotomy. This was explored in detail by Pradeu and Du Pasquier, 2018 who define memory based on five key dimensions, which are all gradients: ‘strength’, ‘speed’, ‘extinction’, ‘duration’, and ‘specificity’. Different forms of memory may fulfil these aspects to varying degrees, that is we cannot say an immune reaction is either ‘with’ or ‘without’ memory; instead, memory can be described along several continua, for example from weak to strong, or unspecific to specific. In the following, we will use immune priming as the more general term (see below) while largely restricting the term memory to cases where at least some of these five key dimensions are fulfilled. In principle, immune memory can thus occur in both innate and adaptive immune systems (Kurtz, 2004; Netea et al., 2019; Sadeghi and Divangahi, 2024). The key criterion distinguishing innate from adaptive immune systems is the occurrence of somatically diversified immune receptors (such as immunoglobulins in vertebrates), which allow the immune system to ‘adapt’ phenotypically over an individuals’ lifetime (Boehm, 2011; Kurtz, 2005). Note that the usage of the word ‘adaptive’ here differs from its meaning in evolutionary biology, where adaptation occurs because of selection over generations. Invertebrate immune systems are generally defined as innate immune systems, although some mechanisms for somatic diversification of immune receptors may exist (see chapter ”Specificity”). Plants do not possess an adaptive immune system and even no specialized immune cells, while showing immune priming phenomena across tissues and cell types that fulfil the criteria of memory to varying degrees (Conrath, 2025; see chapter ”Immune priming in plants”).

Second, we would like to draw attention to the fact that either phenomenological or mechanistic criteria have been used to define memory, which may lead to misunderstandings. For example, vertebrate immune memory has sometimes been defined by the existence of lymphocytes and their products, usually immunoglobulins that result from a somatic recombination process. Such narrow mechanistic definitions may fall short either when memory is found in organisms lacking these mechanisms (Little et al., 2005), or when alternative mechanisms are detected, such as in cyclostomes (jawless fish, such as lamprey), which rely on variable leukocyte receptors but not on immunoglobulins (Boehm and Swann, 2014; Pancer et al., 2004; Pancer and Cooper, 2006), and nevertheless establish immune memory. While we generally prefer phenomenological over mechanistic definitions because of this lack of openness to newly detected mechanisms, we are aware that phenomenological definitions also bear risks, in particular when different aspects of a phenomenon are associated with the same term. For example, the term priming has been used for the exposure to a stimulus, the activation state induced by such stimulus, or the enhanced protection resulting from it, but these terms are not the same (Contreras-Garduño et al., 2016).

Bearing this in mind, we propose a broad, phenomenological definition of the term *immune priming* (Little and Kraaijeveld, 2004). Any kind of changed immune state following a previous encounter with pathogens, pathogen-derived cues, or even other inducers can be termed immune priming. The primed state will usually lead to enhanced protection, that is higher resistance and increased survival upon re-infection, but there may be exceptions. For certain host-pathogen systems, priming may increase tolerance rather than resistance Paraskevopoulou et al., 2022 anisms of transgenerational immune priming; priming may reduce pathogen load, but without increasing host survival, for example Cortacans et al., 2024, or priming may even weaken host resistance (Kutzer et al., 2019). Our definition of immune priming largely follows the concept that underlies the usage of the term in eco-immunology (Little and Kraaijeveld, 2004; Schulenburg et al., 2009). Defining immune priming broadly, based on the phenomenon rather than mechanisms, allows for the inclusion of a vast range of different processes and better represents the diversity of priming phenomena observed across plants and invertebrates (Cooper and Ton, 2022; Lanz-Mendoza et al., 2024; Milutinović and Kurtz, 2016; Prakash and Khan, 2022). Importantly, immune priming may come with memory to varying degrees, based on the definition of memory as a multidimensional and gradual phenomenon as introduced above (Pradeu and Du Pasquier, 2018). Other authors have defined immune priming more narrowly as a nonspecific response that remains elevated and does not return to baseline (Krejčová and Bajgar, 2025). While this may help distinguish it from immune training and immune memory, the problem arises that many phenomena that have been described as priming before would need to be excluded, for example, all those associated with specificity or extinction.

In contrast to the broad definition of immune priming, a narrower definition of *trained immunity* that includes mechanistic aspects could be preferred. According to the definition given by Netea and colleagues (Netea et al., 2020; Netea and Joosten, 2024), trained immunity describes a long-term functional reprogramming of innate immune cells, which is mediated by metabolic rewiring and epigenetic modifications and leads to an altered response towards a second challenge after the return to a non-activated state. Trained immunity provides broad protection beyond the identity of the agent first encountered, that is it is rather unspecific. It can be long-lasting up to several years, but, at variance with ‘classical’ vertebrate adaptive immunity, it can be erased and re-programmed based on the microenvironmental cues-dependent modulation of the epigenetic/metabolic profile. Notably, the accepted definition of trained immunity does not include the phenomenon of innate tolerance that often occurs in mammals, which likewise consists of a long-term functional reprogramming of innate immune cells, based on different metabolic and epigenetic changes, which leads to a different non-specific secondary response after extinction of the primary response. Both trained immunity and some forms of immune priming can be considered alternative forms of innate immune memory, based on the multi-dimensional definition of memory introduced above.

Based on these concepts, certain priming phenomena in invertebrate animals may be caused by trained immunity, if they fulfil the criteria of the specific mechanisms of metabolic and epigenetic reprogramming characterizing trained immunity, that is when they are likely evolutionarily homologous to vertebrate trained immunity. We will explore this possibility further in the following chapters. We will also consider the similarities with acquired immunity in plants, which, despite their lack of innate immune cells, exhibit trained immunity-like responses involving priming resulting from metabolic and epigenetic reprogramming.

## Immune priming in plants

**Figure 1.**
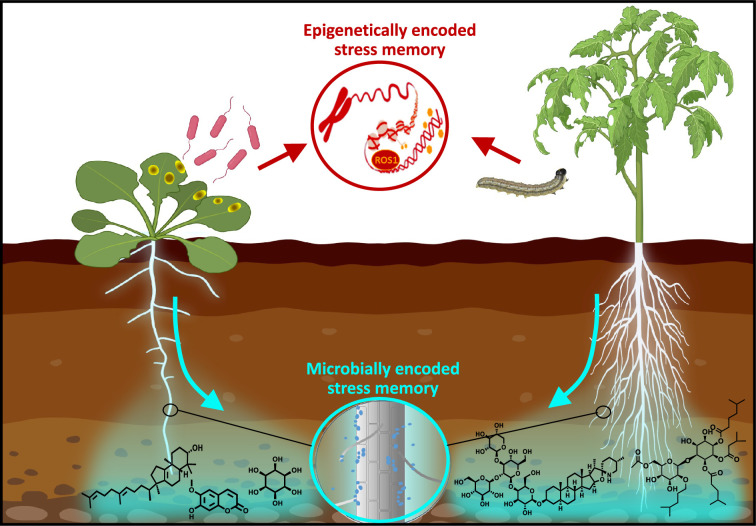
Immune priming in plants. Plants use internal and external mechanisms to generate and maintain defense memory. In response to stress by pathogens or herbivores, plants change the epigenetic makeup of their genome, providing a long-lasting increase in defense capacity via priming of the innate immune system (red). In addition, stressed plants can alter the chemical composition of their root exudates, thereby recruiting and selecting plant-beneficial soil microbes, which in turn can suppress pests and diseases through a range of direct and indirect mechanisms. Together, both strategies provide the plant with long-lasting defense memory, making them more capable of resisting recurrent attacks by pathogens and herbivores.

As primary producers, plants face constant threats from pathogens, herbivores, and abiotic stresses. Despite these pressures, they have successfully colonized and dominated Earth’s terrestrial environments for over 500 million years, a success that can largely be attributed to their multifaceted and versatile ability to defend themselves. Their defensive strategies operate over expanding timescales, ranging from seconds to centuries, and include internal responses at metabolic, epigenetic, and genetic levels, as well as external, ecological responses that rely on the recruitment of plant-beneficial organisms to resist pests and diseases (Wilkinson et al., 2019). A common feature of the long-term strategies is that they equip stress-recovering plants with increased capability to resist recurrent attacks, that is defense memory (Figure 1).

Like other complex multicellular organisms, plants integrate, store, and retrieve information from their environment to anticipate and acclimatize to changing conditions. In animals, the CNS and the cellular immune system provide cognitive and somatic memory functions. Plants lack both systems, yet they show remarkable long-term phenotypic plasticity in response to stress, which helps them to adapt and survive in changing conditions. As animals, plants possess communication systems acting via phytohormones, comparable to the endocrine system and cytokines within the immune system for communication across cells and organs. In addition to the genetically hardwired innate immune system, which offers immediate protection via pattern-triggered immunity (PTI) by microbe- or damage-associated molecular patterns (MAMPs and DAMPs), or effector-triggered immunity (ETI) by specific virulence effectors from pathogens or herbivores (Jones et al., 2024), plants can acquire long-lasting resistance after recovery from pests or diseases. This induced resistance (IR) is typically based on systemic priming of PTI, which mediates a faster and/or stronger defense reaction that offers protection against recurrent attacks by pathogens or herbivores with similar lifestyles (Conrath et al., 2006; De Kesel et al., 2021). Priming is usually less costly than sustained up-regulation of inducible defense mechanisms (van Hulten et al., 2006). However, depending on the strength of the initial stress, priming can also persist throughout the plant’s life cycle and even be transmitted to following generations (Cooper and Ton, 2022).

The classic example of IR by priming comes from systemic acquired resistance (SAR). SAR is triggered in response to an initial innate immune response like ETI or PTI. The induced defense response at the site of exposure then gives rise to long-distance signals that induce and prime defenses in distal plant tissues (Fu and Dong, 2013; Kachroo and Kachroo, 2020). Non-pathogenic triggers of innate immunity, including herbivores or wounding, have also been reported to activate SAR (Kloth and Dicke, 2022; Shah and Zeier, 2013). Additionally, SAR-like responses can also be triggered by insect oviposition and root exposure to incompatible rhizobacteria (Hilfiker et al., 2014; Shine et al., 2019). Induced Systemic Resistance (ISR) is another form of systemic IR, which is typically triggered by beneficial rhizobacteria or mycorrhizae. Although the pathways controlling ISR can vary depending on host-microbe combination, it is typically associated with a priming of ethylene- and jasmonic acid (JA)-dependent defenses (Pieterse et al., 2014) and involves regulation by the iron-deficiency response in the host plant (Stringlis et al., 2018; Trapet et al., 2021). Various chemicals, such as the plant-endogenous stress metabolite β-aminobutyric acid (BABA) and the synthetic compounds benzothiadiazole (BTH, a structural homolog of salicylic acid) and (R)-β-homoserine (RBH), mimic biological IR by priming defense pathways against biotic stresses (Buswell et al., 2018; Cohen et al., 2016; Kohler et al., 2002). These priming agents are effective in a wide range of plant-attacker combinations (Yassin et al., 2021), suggesting common regulatory mechanisms across the plant kingdom. Indeed, the *Arabidopsis* genes encoding the BABA transporter LHT1 (Tao et al., 2022) and BABA receptor IBI1 (Luna et al., 2014b) are highly conserved genes across the plant kingdom and have been implicated in plant responses to both pathogenic and beneficial microbes (Guether et al., 2011; Luna et al., 2014b; Zhang et al., 2022).

While the mechanisms controlling the onset of IR have been studied intensely over recent decades, comparably little is known about the long-term maintenance of IR. SAR efficacy is known to last several months and can be further extended with booster inoculations by the primary immunizer (Bozarth and Ross, 1964; Guedes et al., 1980; Kuć, 1987; Kuc and Richmond, 1977). Thus, long-term maintenance of IR was supposed to involve self-perpetuating resistance-inducing signals that can be maintained through cell division and transmitted into newly formed leaves. Only decades later, research started to focus on the mechanisms underpinning this long-term maintenance of IR. For instance, BABA-induced defense priming against biotrophic pathogens is maintained for at least 4 weeks after induction treatment (Luna et al., 2014a), while a recent study revealed that JA-IR and priming of MYC-dependent defense genes against herbivory is maintained for at least 3 weeks after exposure of seedlings to JA or herbivores (Wilkinson et al., 2023). Both studies also provided evidence for involvement of histone modifications and DNA methylation, supporting the growing notion that long-lasting IR has an epigenetic basis (Hannan Parker et al., 2022). Indeed, multiple studies have reported that plants stressed by pests and diseases produce progeny with enhanced resistance (reviewed in Furci et al., 2023). This heritable IR was first reported by Roberts, 1983, who showed that isogenic progeny from tobacco mosaic virus (TMV)-infected plants develop smaller lesions after challenge with the same virus. Over subsequent decades, evidence emerged that progeny from pathogen- and herbivore-exposed plants display defense-related traits (Agrawal et al., 1999; Holeski, 2007; Molinier et al., 2006), but it was not until the early 2010s that a direct link between heritable IR and priming was established (Kathiria et al., 2010; Luna et al., 2012; Rasmann et al., 2012; Slaughter et al., 2012).

## Immune priming in invertebrate animals

Immune priming has been examined in Porifera, Ctenophora, Cnidaria, Nematoda, Arthropoda, Annelida, Mollusca, Echinodermata, Cephalochordata, and Urochordata (Lanz-Mendoza et al., 2024; Milutinović and Kurtz, 2016). Despite the difficulty in covering further phyla that may be experimentally challenging, demonstration of immune priming in invertebrates led to a paradigm shift, because it is clearly a widespread phenomenon among invertebrates. Immune priming in invertebrates has been reviewed in detail elsewhere (Contreras-Garduño et al., 2016; Lanz-Mendoza et al., 2024; Milutinović and Kurtz, 2016; Milutinović et al., 2016; Sheehan et al., 2020; Sulek et al., 2021), often focussing on insects (Contreras-Garduño et al., 2016; Krejčová and Bajgar, 2025; Sheehan et al., 2020; Sulek et al., 2021). In the following, we thus present key examples demonstrating important milestones and principles to provide a basis for our subsequent focus on certain aspects of priming in the following chapters.

**Figure 2.**
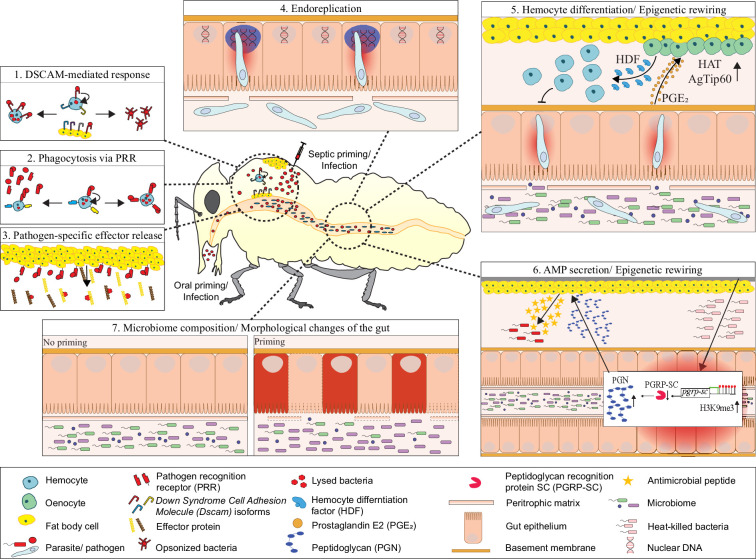
Potential mechanisms of immune priming in invertebrate animals. The figure combines selected examples from different invertebrates, with a focus on arthropods, to show some of the mechanisms involved in immune priming. 1. Dscam acting as a phagocytosis receptor or opsonin mediating specificity in priming Dong et al., 2025; Ng and Kurtz, 2020; Watson et al., 2005; 2. Specificity of phagocytosis based on other pattern recognition receptors (PRRs) Moné et al., 2010; Pham et al., 2007; Roth and Kurtz, 2009; 3. Pathogen-specific effectors differentially regulated upon priming, *e.g*., detected in transcriptome studies Ferro et al., 2019; Tate et al., 2017; 4. Endoreplication in the midgut of mosquitoes upon priming with *Plasmodium* Cime-Castillo et al., 2018; Maya-Maldonado et al., 2021; 5. Midgut epithelial cells release PGE_2_ upon contact with gut microbiota, leading to release of a hemocyte differentiation factor (HDF) from oenocytes, which is dependent on Tip60-mediated histone acetylation Gomes et al., 2021; 6. Epigenetically controlled expression of PGRP-SC in the gut mediating AMP secretion by the fat body Deng et al., 2025; 7. Oral priming may depend on the presence of microbiota (Futo et al., 2015; Prakash et al., 2023), lead to changes in microbiota composition (Korša et al., 2022) and gut morphology (Baur et al., 2025). Figure based on Milutinović et al., 2016 with updates informed by recent literature.

Immune priming as a protective mechanism was initially described by McKay et al., 1969, who showed that the crayfish *Parachaeraps bicarinatus* previously challenged with killed *Pseudomonas* bacteria exhibited lower mortality to a subsequent lethal challenge of the same bacteria, in comparison to a control group without prior immunization. Such ‘vaccination’ also worked with a few other tested Gram-negative, but not with Gram-positive bacteria, thereby showing a certain degree of specificity. Likewise, Boman et al., 1972 showed that immunization of *D. melanogaster* with *Arthrobacter cloacae* reduced growth of *A. cloacae*, as well as *P. aeruginosa* and *Escherichia coli*, upon secondary infection. In the following decades, studies in further invertebrate taxa provided similar evidence. Combined with studies on allograft rejection in invertebrates, these observations not only gave rise to the field of comparative immunology (Cooper, 2018), but also raised intensive discussions in the 1980s and 1990s regarding their controversial interpretation (Cooper et al., 1992; Klein, 1989; Klein, 1997). Are these observations based on homology, and should they be called immune memory or not? Overshadowed by the fruit fly *Drosophila melanogaster* that had emerged as an extremely powerful model for studying the genetics of innate immunity in the late 1990 (Hoffmann and Reichhart, 2002), further studies accumulated evidence that invertebrate immune responses exceeded what could be expected from purely innate defenses. That immune priming could attain non-specific protection was proposed by Moret and Siva-Jothy, 2003, who showed that insects injected with lipopolysaccharides were more resistant against a subsequent infection with a fungus. The field gained momentum in 2003 with the demonstration of an unexpectedly high degree of specificity in the defense of a crustacean against repeated exposures by a natural tapeworm parasite (Kurtz and Franz, 2003). In the following years, the demonstration of such ‘specific immune priming’ further accumulated (Sadd and Schmid-Hempel, 2006) and included insect model species such as *Drosophila* and *Tribolium* (Contreras-Garduño et al., 2015; Pham et al., 2007; Rodrigues et al., 2010; Roth et al., 2009), which helped provide some insights into its mechanistic underpinnings (Figure 2). A range of effector molecules associated with the immune response (both evolutionary conserved and taxon-specific), metabolism-related molecules, epigenetic mechanisms, and cellular endocycling have been identified (Lanz-Mendoza et al., 2024; Méndez-López et al., 2024; Mukherjee and Dobrindt, 2024). However, in most systems, it remains largely unclear how homologous and heterologous challenges are specifically distinguished, which cells are involved, how memory is stored following recognition, and how subsequent recall and elimination are carried out (Lanz-Mendoza and Contreras-Garduño, 2022). The impression is solidifying that invertebrate immune priming is a widespread phenomenon, and that the mechanisms leading to it are as diverse as invertebrates as a group (Milutinović and Kurtz, 2016). While some mechanisms underlying immune priming may be restricted to certain taxa, others might be more universal, such as the involvement of phagocytic cells (Pham et al., 2007; Roth and Kurtz, 2009; Figure 2). Mechanisms that are functionally equivalent to processes observed in the vertebrate immune system may be mediated by other cell types. For example, although invertebrates lack neutrophils, immune responses functionally equivalent to neutrophil extracellular traps (NETs; Brinkmann et al., 2004)—which are also implicated in trained immunity (Gao et al., 2022)–are mediated by invertebrate hemocytes (Ng et al., 2013) and appear to contribute to immune priming (Cho and Cho, 2024).

On the other hand, it turned out that immune priming may not be universally observed, and that in some cases natural enemies (such as the entomopathogen *Bacillus thuringiensis*; Ferro et al., 2019) may be more likely to elicit it. Conversely, sometimes even non-pathogenic elicitors can induce a primed state, for example a temperature shock (Eggert et al., 2015). Exposure to an environmental stress (hypoxia and starvation) can even differentially prime different organs in the same animal (*Ciona robusta*), resulting in a distinct immune gene profile in the gut and in the pharynx, the two main immune-related organs, upon a secondary challenge (Marino et al., 2023), an observation that underlines the plasticity of immune priming in adapting the reactivity of each organ in the body to the future challenges.

Immune priming in invertebrates may also occur across generations (so-called transgenerational immune priming, TGIP): parents can specifically protect their offspring (and occasionally grand offspring) against infections (for review, see Rutkowski et al., 2023; Vilcinskas, 2021). For example, Wilson et al., 2021 tested transgenerational immune priming in *Spodoptera exempta* against a dsDNA virus. Their results demonstrated protection from parents to offspring that was dependent on the dose of the initial challenge in the parents: those challenged with low viral doses could transfer priming to the offspring, while no priming was observed in the progeny of parents challenged with high viral doses. When adult *Drosophila* are cohabited with a parasitic wasp, they produce offspring that is more capable of mounting a successful immune response (Bozler et al., 2020). In *T. castaneum,* TGIP affects bacterial growth dynamics in offspring (Tate et al., 2017), and both mothers and fathers provide protection to their offspring against bacterial challenges (Roth et al., 2009). Consistent with this, a recent meta-analysis corroborates the existence of transgenerational immune priming and indicates that parents provide a similar protection to their offspring (Rutkowski et al., 2023). Nonetheless, like in the case of within-generation priming, there remains a significant gap in our understanding of the mechanisms underlying TGIP (Prakash and Khan, 2022; Rutkowski et al., 2023).

## The innate immune memory in vertebrates: trained immunity and tolerance

Vertebrates display highly specific immune mechanisms (adaptive immunity) that complement the ancient, conserved mechanisms of immunity present in invertebrates, which in vertebrates are collectively defined as ‘innate immunity’. The presence of the specific adaptive immunity has probably led to a compartmentalization of functions between the two systems and has limited the specificity of innate immunity, a specificity that is evident in invertebrate immunity. However, the innate immune memory, that is the capacity of the innate immune system to ‘remember’ a previous stimulus, is retained in vertebrates, although with some subphylum-specific differences. A phenomenon ascribable to innate memory has been described in mammals by Paul Beeson in 1946. He wanted to explain the mechanisms of tolerance observed in human patients subjected to fever therapy, which occurred after multiple intravenous administrations of a typhoid vaccine (fever therapy was used until the ’40 of the last century as the sole treatment for diseases such as syphilis, before the introduction of penicillin). Beeson repeated the treatment in rabbits and reproduced the establishment of tolerance (no fever induction) upon repeated administration of killed typhoid bacteria and demonstrated that the reticuloendothelial system (i.e. macrophages) was responsible for such tolerance (Beeson, 1946; Beeson, 1947). This kind of tolerance, induced by bacterial challenges, is a bona fide innate memory phenomenon that implies a complex cellular reprogramming, resulting in a reduction of inflammatory responses together with an increase of anti-inflammatory mechanisms (Buckley et al., 2006; Cavaillon and Adib-Conquy, 2006; Fan and Cook, 2004). Many other studies followed Beeson’s pioneering work, mostly in mice and mostly describing the tolerance type of innate immune memory displayed by macrophages and the macrophage-dependent establishment of non-specific protection after in vivo priming with microorganisms (Bistoni et al., 1986; Boraschi and Meltzer, 1979; Dubos and Schaedler, 1956; Foster et al., 2007; Freudenberg and Galanos, 1988; Seeley and Ghosh, 2017; Youmans and Youmans, 1965). In vertebrates, another innate memory phenomenon was described, similar to invertebrates, that is an increased innate/inflammatory reactivity upon a second challenge. This phenomenon was dubbed ‘trained immunity’ by Mihai Netea in 2011 (Ifrim et al., 2014; Netea et al., 2016; Netea et al., 2011; Quintin et al., 2014). Both phenomena, tolerance and trained immunity, aim at improving the host response to future challenges, either by the reduction of an excessively strong reactivity, as in the case of the endotoxin tolerance firstly described by Beeson, 1946, or by enhancing the defensive innate response to the new challenge. Trained immunity implies the increased secondary production of inflammatory cytokines (typically TNFa and IL-6) by monocytes and macrophages after a previous exposure to some microbial or endogenous agents (BCG, b-glucan, oxLDL), evident in vitro but also in vivo in mice and humans, other mammals and non-mammalian vertebrates (fish, birds; Angulo and Angulo, 2022; Guerra-Maupome et al., 2019; Paris et al., 2020; Petit et al., 2019; Verwoolde et al., 2021; Vierboom et al., 2021). Other cells, besides myeloid cells, can display trained immunity, such as epithelial cells, fibroblasts, and innate lymphoid cells (Cassone, 2018; Hamada et al., 2018; Hammer and Romagnani, 2017; Naik et al., 2017), a fact that underlines the broadness of this protective mechanism.

Both trained immunity and tolerance are mainly based on epigenetic and metabolic changes (Domínguez-Andrés et al., 2020; Logie and Stunnenberg, 2016; Netea et al., 2016; Novakovic et al., 2016; Saeed et al., 2014; Sun and Barreiro, 2020; van der Heijden et al., 2018). Histone and DNA methylations and histone acetylations have been described to occur during establishment of trained immunity, while different epigenetic and bioenergetic shifts appear to correlate with tolerance (Domínguez-Andrés et al., 2020; Logie and Stunnenberg, 2016; Novakovic et al., 2016; Saeed et al., 2014; Sun and Barreiro, 2020; van der Heijden et al., 2018). A role in innate memory has been reported also for non-coding RNA, both lncRNAs and miRNAs (Fanucchi and Mhlanga, 2019; Seeley et al., 2018). However, there is no clear evidence in vertebrates for a role in innate memory by RNA interference, which is a major anti-infective mechanism across evolution (Boraschi et al., 2024; Guo et al., 2019) and that is a main memory phenomenon in invertebrates. Regarding energy metabolism, trained immunity appears to be linked to enhanced glycolysis, whereas tolerance correlates with enhanced mitochondrial oxidative phosphorylation (Ferreira et al., 2022; Italiani et al., 2020).

The duration of innate memory in vertebrates is still a matter of speculation. Theoretically, the persistence of innate memory mechanisms based on epigenetic modifications can go well beyond the lifespan of the primed cells, because epigenetic modifications can be transmitted to daughter cells and induced in hematopoietic progenitors in the bone marrow microenvironment (Mitroulis et al., 2018). On the other hand, epigenetic modifications can be rapidly erased in response to microenvironmental changes and/or replaced by different modifications, suggesting that the duration of epigenetic memory strongly depends on the dynamics of the microenvironmental cues. In this respect, it should be noted that neurons of the insular cortex can sense peripheral innate/inflammatory reactions (immunoception) and store immune representations (immunengrams), which may then retrieve or suppress a subsequent peripheral innate/inflammatory response (Koren et al., 2021; Rolls, 2023). This suggests that trained immunity and tolerance are not exclusively based on epigenetic and metabolic reprogramming of the immune cells, and that innate memory generation and maintenance is regulated by a complex network of endogenous and exogenous stimuli, so as to afford the best protective efficacy in a constantly changing environment.

Finally, it is important to note that, in mammals, there is evidence of organ-selective immune memory. Immunity in different mammalian organs shows different characteristics for an optimal adaptation to the specific functions and needs of the organ. The diversity of tissue-resident macrophages is exemplary (Kupffer cells in the liver, Langerhans cells in the skin, alveolar macrophages in the lung, osteoclasts in the bone, microglial cells in the brain) and underlines the importance of the diverse organ microenvironments for the development of optimal immunological functions. In this context, it is expected that the innate memory generated in a particular tissue compartment, in response to an organ-specific infection and to a number of concomitant microenvironmental cues, could preferentially induce enhanced protection within that organ rather than in the entire organism. This indeed appears to be the case. Thus, memory generated in the lung by respiratory infections is more protective at the lung level and against lung challenges (Cauchi and Locht, 2018; Lee et al., 2018; Rasid and Cavaillon, 2018; Yao et al., 2018), and the brain-engraved memory of gut inflammation can specifically retrieve a gut inflammatory reaction (Koren et al., 2021). Although this is not a specific memory as intended in adaptive immune responses, it is evident that innate memory can be specifically shaped by exogenous (infectious agents) and endogenous (tissue-related) stimuli to afford enhanced protection in restricted tissues and against a restricted number of pathogens.

## Epigenetic and metabolic processes in immune priming

Epigenetic mechanisms play a crucial role in regulating immune priming in plants and invertebrates, as well as trained immunity in mammals, despite the differences in scope and specificity (Mukherjee and Dobrindt, 2024). Epigenetic mechanisms modify gene expression without altering the underlying DNA sequence. This is primarily achieved through changes in chromatin structure, which influence the accessibility of transcription factors to DNA, affecting gene expression. DNA methyltransferases (DNMTs) catalyze DNA methylation, which in animals occurs mainly at CpG sites and is maintained by DNMT1, established by DNMT3, while DNMT2 instead methylates tRNA; RNA can also be methylated at various positions. Beyond DNA and RNA, histone modifications such as acetylation and methylation strongly influence gene expression and chromatin structure, but their roles in innate immune memory are only beginning to be understood. When it comes to the potential role of epigenetics for immune priming, it is important to note that the specific functions of epigenetic modifications in invertebrates may differ substantially from the well-known functions in vertebrates. For example, while promoter DNA methylation in vertebrates is linked to repression of gene expression, it mostly occurs within gene bodies in insects, with largely unknown function (Engelhardt et al., 2022; Glastad et al., 2019; Zemach et al., 2010). Different epigenetic modifications are interconnected, and multi-omics studies are needed to further clarify their specific functions in the species of interest (Länger et al., 2025) but have to our knowledge not been conducted in relation to immune priming phenotypes.

**Figure 3.**
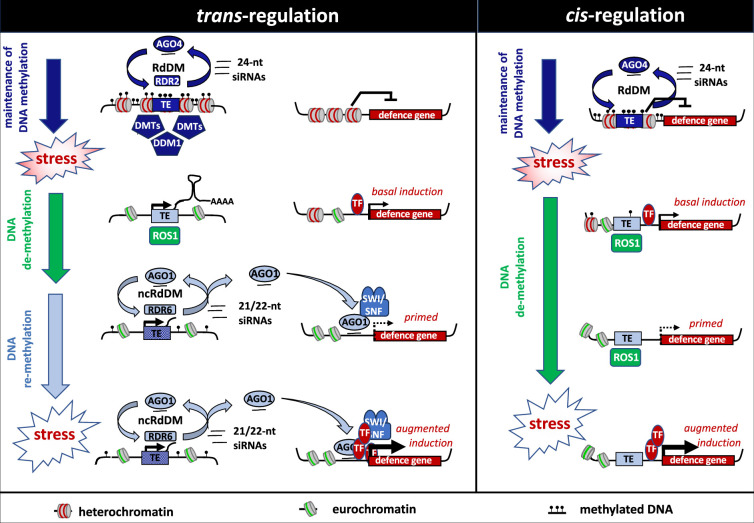
Two models of epigenetically controlled immune memory in plants. The *trans*-regulatory model involves major induced DNA de-methylation in TE-rich peri-centromeric regions by ROS1, which antagonizes the heterochromatin maintenance machinery by DNA methyltransferases (DMTs), chromatin re-modelers (e.g. DDM1) and canonical RNA-directed DNA methylation (RdDM). To prevent large-scale pericentromeric TE expression and nuclear disruption, post-translational gene silencing and RDR6-dependent non-canonical RdDM (ncRdDM) rapidly generate 21/22-nt small RNAs (sRNAs) that are loaded onto AGO1. Due to partial sequence complementarity between TEs and distant defense genes, sRNAs-AGO1 complex interacts with gene bodies/promoters of distant defense genes and recruits the SWI-SNF chromatin re-modeling complex, which relaxes the chromatin environment and primes the defense genes for enhanced induction by transcription factors (TFs) upon secondary stress. The *cis*-regulatory model on the right involves stress-induced DNA demethylation of TEs near defense genes within the chromosome arms. Due to a delayed DNA re-methylation response in these regions, the relaxed chromatin environment of the hypo-methylated TE mediates long-term priming of the nearby defense gene. The *trans*-model is based on Liu et al., 2018 and Wilkinson et al., 2023; the *cis*-model is based on López Sánchez et al., 2016 and Halter et al., 2021.

Immune priming in plants (Figure 3) involves a complex interplay between various epigenetic mechanisms, including DNA (de)methylation, small RNAs, histone PTMs, and histone variants (Hannan Parker et al., 2022). Histone PTMs influence chromatin density and gene expression and can be maintained through cell division (Zhao et al., 2019). The first evidence for involvement of epigenetic mechanisms in plant immune priming came from reports that euchromatic histone PTMs, such as H3K4me3 and H3K9ac, are enriched in promoters of primed defense genes (Jaskiewicz et al., 2011; Luna et al., 2012). More recently, mRNA- and FAIRE-sequencing analysis of SAR-expressing leaves revealed that promoter elements of primed defense genes are enriched with open chromatin sites (Baum et al., 2019), which facilitates the access of the transcriptional machinery upon secondary challenge, resulting in a faster and/or stronger transcriptional induction. Changes in DNA methylation have also been implicated in plant immune priming. DNA methylation within plant genomes primarily serves to silence expression of deleterious transposable elements (TEs) and occurs at CG, CHG, and CHH contexts, with its establishment and maintenance controlled by interdependent pathways (Gallego-Bartolomé, 2020). Unlike histone PTMs, plants do not completely reset DNA methylation patterns acquired over their lifetimes (Quadrana and Colot, 2016), allowing for transmission of epigenetically encoded information to subsequent generations. This is supported by reports that stably inherited regions with reduced DNA methylation in TE-rich regions of isogenic epigenetic recombinant inbred lines (epiRILs) confer high disease resistance by genome-wide defense gene priming (Furci et al., 2019). Furthermore, independent studies have reported that biotic stress in plants leads to DNA de-methylation, providing a plausible mechanism by which IR is maintained and transmitted to following generations. Indeed, *Arabidopsis mutants* with reduced DNA methylation display innately primed defense phenotypes (Luna et al., 2012; Rasmann et al., 2012), whereas hypermethylated mutants in the DNA demethylase ROS1 show repressed PTI responsiveness and increased susceptibility to pathogens and are impaired in heritable IR (Halter et al., 2021; López Sánchez et al., 2016; Yu et al., 2013a). A recent study, furthermore, revealed that ROS1 enhances the epimutation rates of genes with low steady-state DNA methylation and high levels of H3K27me3 and histone variant H2A.Z under multigenerational disease pressure (Zhang et al., 2024a). Exactly how ROS1-dependent DNA demethylation primes defense genes remains open for debate (Cooper and Ton, 2022). While ROS1 has been shown to *cis*-regulate SA-dependent defense genes by targeting W-box elements in their promoters (Charvin et al., 2023; Halter et al., 2021), there is also evidence that DNA de-methylation of TE-rich peri-centromeric regions primes distant defense genes via *trans*-regulatory mechanisms (Cambiagno et al., 2018; Furci et al., 2019). A recent study by Wilkinson et al., 2023 reported that immune memory of JA-dependent immunity against herbivores is regulated in trans by ROS1-targeted TEs, which prime distant MYC-dependent defense genes via 21/22-nt small RNAs (sRNAs) and the RNA-binding protein AGO1. AGO1 and its role in sRNA biogenesis have also been shown to regulate SAR (Shine et al., 2022). Future research is needed to explore how epigenetic interactions between TE-rich pericentromeric regions and gene-rich chromosome arms control the onset, maintenance, and erasure of stress-specific immune memory in plants.

In insects, epigenetic changes could facilitate primary innate immune response occurring on the first exposure to a pathogen. For example, bacterial infection in the cotton bollworm *Helicoverpa armigera*, fruit fly *D. melanogaster,* or the mosquito *Aedes aegypti* is linked to the stimulation of DNMT gene expression (Baradaran et al., 2019; Bhattacharya et al., 2017; LePage et al., 2014). Similarly, in the greater wax moth *Galleria mellonella,* immune gene expression after infection with the entomopathogenic fungus *Metarhizium robertsii* is associated with altered HAT and HDAC activities (Mukherjee et al., 2012). Epigenetic changes also occur in *Anopheles gambiae* mosquitoes during an enhanced immune response after *Plasmodium* ookinete invasion that allows direct contact between the gut microbiota and midgut epithelial cells by disrupting the peritrophic matrix, resulting in immune priming. The production of hemocyte differentiation factor (HDF), which is a key component of immunity against *Plasmodium* of the immune-primed *A. gambiae*, is dependent on activity of HAT Tip60 (Gomes et al., 2021). Immune priming in the mealworm beetle *Tenebrio molitor* was associated with reduced RNA methylation compared to non-primed counterparts after challenge with the bacterium *Micrococcus lysodeikticus* in adults or with the fungus *Metarhizium anisopliae* in larvae (Castro-Vargas et al., 2017).

Multiple mechanisms, including epigenetic processes, are involved in immune priming at the intergenerational and transgenerational levels (Freitak et al., 2014; Milutinović and Kurtz, 2016; Mukherjee and Dobrindt, 2022). For instance, feeding the parental generation of the tobacco hornworm (*Manduca sexta*) with the entomopathogenic bacterium *Serratia entomophila* led to significant downregulation of HDAC4 and HAT Chameau, but only in female offspring (F1 generation; Gegner et al., 2019). Similarly, parental exposure to *E. coli* induced sex-specific differences in the expression of DNMT1 and DNMT2 in F1 larvae (Gegner et al., 2019). DNA methylation level was reduced in the F1 generation in male and female offspring of both parents in both treatment groups (*S. entomophila* vs. *E. coli*). Further, in the red flour beetle *T. castaneum*, higher *Dnmt2* expression in male testes suggests that DNMTs may interfere with paternal immune transfer (Schulz et al., 2022). RNAi knockdown of *Dnmt2* in fathers slowed offspring development and increased adult mortality after *B. thuringiensis* infection but did not affect *Dnmt2* expression or selected innate immune genes in the offspring (Schulz et al., 2022). Repeated exposure of *G. mellonella* larvae to *B. thuringiensis* or *M. robertsii* over generations increased infection resistance alongside tissue-specific changes in DNA methylation and histone acetylation (Mukherjee et al., 2019; Mukherjee et al., 2017).

The described epigenetic processes are linked to cellular metabolism that provides the substrates needed for epigenetic modifications. Accordingly, changes in metabolism and epigenetics co-occur during vertebrate trained immunity and might even be directly connected (Ziogas et al., 2025). Knowledge about metabolism is more limited for invertebrate immune priming, but hints into a similar direction. In insects, infection triggers metabolic changes, such as increased glycolysis and tricarboxylic acid (TCA) cycle activity, which boost the energy production necessary for a robust immune response (González‐Santoyo and Córdoba‐Aguilar, 2012). In mosquitoes (*Anopheles albimanus*), transcripts involved in carbohydrate metabolism, TCA cycle, lipid metabolism, and fatty acid synthesis show differential expression during the induction of immune priming. Transcripts identified as trehalose transporter, GDP-D glucose phosphorylase, and fatty acid hydroxylase are upregulated only in primed conditions (Maya-Maldonado et al., 2021). These metabolic adaptations may enhance the mosquito’s immune response upon subsequent pathogen encounters. Increased energy production and synthesis of immune components during immune priming are regulated by eicosanoids and juvenile hormones, although these mechanisms involve a trade-off between reproduction and immunity (Contreras-Garduño et al., 2014; Contreras-Garduño et al., 2016; Khan et al., 2019). Moreover, metabolic pathways can amplify immune gene expression during priming by interacting with epigenetic pathways and chromatin-modifying enzymes.

So far, only a few studies have directly addressed the question as to whether the interaction of epigenetic regulation with metabolic reprogramming of immune cells that characterizes vertebrate trained immunity (Arts et al., 2016; Gomes et al., 2021) could also be detected in invertebrate immune priming. Although indirect evidence points towards such a connection (Ferro et al., 2019; Tate et al., 2017), a review provides limited direct proof for a link between epigenetic regulation and metabolism in arthropod and mollusc immune priming (Zhao et al., 2023). Zhao et al., 2024 recently reported that increased glycolysis upon secondary challenge with the bacterium *Aeromonas hydrophila* is regulated via lysine demethylase 4 homologue (*KDM4*)-mediated regulation of H3K9me3-modifications at promoters of the genes *PFK* and *G-6-PD* (which are key enzymes of glycolysis and the pentose phosphate pathway, respectively) in hemocytes of the crab *Eriocheir sinensis*. In *Drosophila*, gut-mediated bacterial immune training involves systemic priming in the fat body due to reduced expression of the peptidoglycan recognition protein PGRP-SC, mediated via increased promoter histone methylation (H3K9me3; Deng et al., 2025; Figure 2). Moreover, hemocyte metabolic reprogramming in *Drosophila* has recently been shown to not only resemble similar metabolic processes in vertebrate macrophages, but to even affect developmental plasticity of the whole organism (Mahanta et al., 2025). Together, these studies point towards a metabolism-epigenetics link in invertebrate immune priming but await functional proof.

Taken together, both immune priming and trained immunity emphasize the importance of epigenetic processes in regulating immune responses. Whether through DNA methylation, histone modifications, or other mechanisms, these processes offer a dynamic and reversible means of immune regulation, providing organisms with an evolutionary advantage in their defense against pathogens. Understanding these mechanisms may open new avenues for immunotherapy, vaccination strategies, and even pest control by harnessing the power of epigenetic reprogramming in both insects and vertebrates. Future research that explores the longevity and heritability of epigenetic changes in insects and humans could reveal new mechanisms, potentially bridging innate and adaptive immunity and bringing us closer to a unified understanding of immune memory across species.

## Signaling processes in immune priming in plants and invertebrates

Plants and invertebrates exhibit immune memory generated in response to the activation of PAMP- (invertebrates, plants) or effector- (plants) triggered immunity despite the lack of lymphocytes and an adaptive immune system. Furthermore, the lack of a circulatory system does not prevent the systemic manifestation of immune memory in plants. One example is SAR, which is induced in response to primary infection and provides broad-spectrum and long-lasting protection against subsequent infections (Chester, 1933; Kachroo and Kachroo, 2020; Kuć, 1987). Notably, volatile emissions from SAR-activated plants were shown to enhance pathogen resistance in neighboring plants suggesting that immune signals can be transferred between neighboring individuals (Riedlmeier et al., 2017; Wenig et al., 2019).

During SAR, rapidly generated and systemically transported (within 4–6 hr of primary infection) signal(s) prime the uninfected parts of the plant against future infections (Chanda et al., 2011; Kachroo and Kachroo, 2020; Rasmussen et al., 1991). Thus, SAR signal(s) generation, their systemic transport and perception in the distal tissue are all essential aspects of SAR signaling. Intense focus on identifying the mobile signal(s) has revealed that the base SAR signaling pathway operates via a dual-branched process with additional non-linear interactions amongst some constituents (Bernsdorff et al., 2016; Chanda et al., 2011; Wenig et al., 2019; Yu et al., 2013b). One branch comprises salicylic acid (SA; Vlot et al., 2009) and its signaling component NPR1 [Nonexpressor of *Pathogenesis-Related* (*PR*) genes] (Dong, 2004). SA accumulation results in NPR1 translocation from the cytosol to the nucleus where it interacts with TGA (TGACG motif binding) transcription factors (Després et al., 2000; Spoel and Dong, 2024; Zavaliev and Dong, 2024) resulting in target gene expression (Wang et al., 2006; Wang and Dong, 2011). NPR1 is also required for transgenerational transfer of SAR (Jaskiewicz et al., 2011; Luna et al., 2012). While there is no evidence to suggest that SA accumulation (above basal levels) in the primary infected tissue is essential for SAR, SA does consistently accumulate in the distal tissue, and this likely contributes to priming, resulting in stronger responses to subsequent infections (Gruner et al., 2013; Návarová et al., 2012). A recent study showed that hydrogen peroxide-mediated sulfenylation of the CHE transcription factor (CCA1 Hiking Expedition) increases SA accumulation in the systemic tissue by promoting the expression of the SA-synthesis gene *ICS1* (Cao et al., 2024). Notably, some percentage of SA is always transported from the primary infected tissue to the distal tissues (Kiefer and Slusarenko, 2003; Meuwly et al., 1995). While intracellular movement of SA occurs via the apoplast (space between the cell wall and plasma membrane), long-distance transport involves the plant cuticle (hydrophobic barrier surrounding all aerial surfaces of the plant) (Lim et al., 2020; Lim et al., 2016). Defects in the cuticle increase transpiration and thereby reduce water potential, which in turn reduces apoplastic transport of SA. It is possible that distal transport of SA also involves derivatization of SA to its volatile and phloem mobile derivative MeSA (methyl SA), because the conversion of SA to MeSA and back to SA is critical for SAR (Chen et al., 2003; Effmert et al., 2005; Koo et al., 2007). The precise relationship between SA and MeSA in SAR needs further clarification.

The other branch of SAR comprises pipecolic acid (Pip; Návarová et al., 2012; Wang et al., 2018), nitric oxide (NO), and reactive oxygen species (ROS; Wang et al., 2014), azelaic acid (AzA; Gao et al., 2015; Jung et al., 2009; Yu et al., 2013b), and glycerol-3-phosphate (G3P; nal Priming of Offspring Immune Syst Chanda et al., 2011). G3P regulates the stability of trans-interfering small RNA *TAS3a* (Shine et al., 2022), which also serves as an early mobile signal of SAR. During SAR, G3P and AzA are intracellularly transported via the plasmodesmata (PD), and this requires the PD localizing protein 5 (Lim et al., 2016). SA accumulation inhibits G3P and AzA transport by regulating PD aperture (Lim et al., 2016). G3P and AzA also require the non-specific lipid transfer-like protein (LTP), DIR1 (defective in induced resistance), and the hybrid proline-rich protein, AZI1 (AzA insensitive), to function as SAR inducers (Cecchini et al., 2015; Jung et al., 2009; Lim et al., 2016). DIR1, AZI1, and its paralog EARLI1 (Early *Arabidopsis* Aluminium Induced 1) interact with each other, and all three proteins are essential for SAR (Cecchini et al., 2015; Chanda et al., 2011; Yu et al., 2013b). Additional SAR regulators include dehydroabietinal (DA; Chaturvedi et al., 2012), *N*-hydroxy Pip (NHP; Chen et al., 2018; Hartmann et al., 2018), and extracellular (e)NAD(P) (Wang et al., 2019). Pinene volatiles are involved in immune signal transfer amongst individual plants and function downstream of a positive feedback loop between Pip and G3P, supporting additional non-linear interactions amongst some of these regulators (Riedlmeier et al., 2017; Wenig et al., 2019). An understudied aspect of SAR signaling that warrants further investigation pertains to the processes underlying signal perception in the distal tissue. Although the cuticle was shown to be important for this process (Xia et al., 2009), subsequent studies showed that the severity of cuticular damage and/or additional unknown factors may regulate SAR signal perception (Xia et al., 2012).

In invertebrate animals, the signaling pathways involved in immune priming are poorly understood. However, there is evidence that several immune pathways are activated during priming and after a second contact with the pathogen. In the sea anemones, *Exaiptasia pallida,* animals that had previously encountered the pathogen *Vibrio coralliilyticus* under sub-lethal conditions had a higher survival upon a subsequent lethal challenge, compared to naive anemones that encountered the pathogen for the first time (Brown and Rodriguez-Lanetty, 2015). Authors found that several proteins involved in stress response were detected in association with defense priming, particularly a putative heat shock protein 70 (HSP70) ortholog. The role of this protein in the activation and maintenance of immune memory is not clear. Still, it opens the possibility that HSP70 can activate immune response through activating TLRs and NF-kB factors translocation.

One of the critical pathways involved in immune priming in molluscs is the Toll-like receptor (TLR) signaling pathway. Research indicates that TLRs are crucial in pathogen recognition and the subsequent immune response in various mollusc species. For instance, studies have shown that upon secondary exposure to pathogens, such as *Vibrio* species, the expression of TLR signaling molecules is significantly upregulated, suggesting a primed immune response (Yao et al., 2021; Zhang et al., 2024b). Similarly, enhanced expression of *Tlr3*, *Myd88-2,* and *Il17-1* was reported specifically in the hemocytes of the immune-primed Pacific oyster *Crassostrea gigas* (Lian et al., 2024; Wang et al., 2020).

In insects, classical intracellular immune pathways such as Toll, IMD, and JAK-STAT likely regulate immune priming. These pathways may protect against recurring infections by triggering the production of antimicrobial peptides (AMPs), reactive oxygen species (ROS), and other immune defense molecules. For example, honeybees fed with heat-killed pathogens exhibit immune priming via Toll-mediated upregulation of defensin-1 and other immune genes (Kim et al., 2023). In *D. melanogaster*, the Toll pathway and phagocytes are involved in immune priming against Gram (+) *Streptococcus pneumoniae* (Pham et al., 2007), whereas the IMD pathway is involved in priming against Gram (-) *Providencia rettgeri* (Prakash et al., 2024). Besides Toll and IMD, the JAK-STAT pathway–which is crucial for antiviral immunity in insects–primes the immune system for future viral challenges, such as those posed by the dengue virus in mosquitoes (Mushtaq et al., 2024). Additionally, in *Caenorhabditis elegans,* immune priming was dependent on the insulin signaling (DAF-16) and p38 MAP kinase (PMK-1) pathways (Anyanful et al., 2009).

Invertebrates also employ extracellular immune pathways to regulate immune priming. In *Anopheles gambiae*, prostaglandin E2 (PGE2), released from the midgut enhances the patrolling activity of hemocytes by attracting them to the midgut surface, resulting in a more robust immune response during re-infection (Barletta et al., 2019; Figure 2). Tassetto et al., 2017 described a novel RNAi amplification and dissemination mechanism, where hemocytes take up dsRNA from infected cells and produce virus-derived complementary DNAs (cDNA) using a transposon-encoded reverse transcriptase. These cDNAs provide a template of de novo synthesis of secondary viral siRNA (vsRNA), secreted in exosome-like vesicles, conferring passive protection against virus challenge in naive animals.

**Figure 4.**
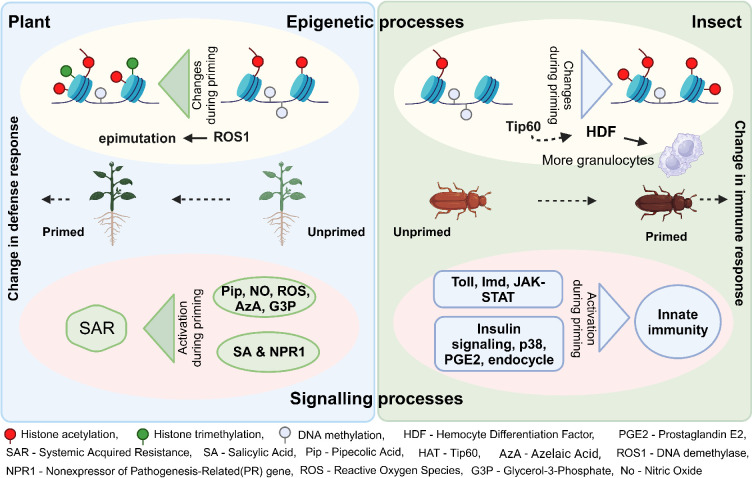
Signaling and epigenetic processes. The figure provides a simplified overview of some of the key mechanisms involved in immune priming in plants and invertebrates. Created with BioRender.

In mosquitoes, epithelial cells are suggested to play a crucial role in establishing immune priming associated with the endocycle (Contreras-Garduño et al., 2015; Maya-Maldonado et al., 2022). In *A. albimanus*, priming with *Plasmodium berghei* resulted in a significant upregulation of cell cycle element genes, and factors activating the endoreplication pathway like Notch and Hnt were observed upon subsequent challenge (Maya-Maldonado et al., 2022; Figure 2). In the same model system, the immune memory group showed more cellular activity of the endocycle than the control group (Contreras-Garduño et al., 2015; Maya-Maldonado et al., 2022). Similar results have been obtained in *Ae. aegypti* mosquitoes, implicating the endocycle in invertebrate immune memory. It is also interesting that endoreplication and the Notch pathway are activated following trained immunity in monocytes (Cime-Castillo et al., 2018). The molecular basis of these pathway activations and their relationship to immune priming in insects remains to be determined. It will also be fundamental to extend these studies to further invertebrate groups. Although, in invertebrates, pathways related to epigenetics, endocycle, oxidative stress, and metabolism occur after the first and the second challenge, there is no information about potential differences between specific *versus* non-specific immune protection (Méndez-López et al., 2024). Figure 4 provides a simplified comparison of selected signaling and epigenetic processes involved in immune priming in plants and invertebrates.

## Specificity

Specificity is a hallmark of vertebrate adaptive immunity, where we often speak of ‘specific immune memory’. More generally, immune specificity can be defined as the degree to which immune reactions differentiate between antigens. It is opposed to cross-reactivity, which measures the extent to which different antigens appear similar to the immune system. Pradeu and Du Pasquier, 2018 consider specificity to be one of five critical dimensions of immunological memory. Kurtz, 2005 even puts specificity into the center (next to induction) when defining specific immune memory.

On closer inspection, immunological specificity is not a simple concept (Vivier and Malissen, 2005). For example, the existence of highly specific immune receptors does not necessarily mean that the immune response as a whole is specific: the ligand recognized by an immune receptor could be shared by many pathogens (as is the case for many innate immune receptors), or the effector non-specifically attacks many different pathogens (as is the case for many innate immune reactions). In both cases, at a phenotypic level, the immune reaction may appear to be unspecific, even though the involved receptors recognize antigens with high specificity. The extremely high specificity provided by vertebrate adaptive immune systems is based on the large number of receptors (produced by somatic diversification) combined with the fact that the receptors are closely coupled to effector specificity, as in the case of antibodies and B- and T-cell receptors.

In invertebrate animals, immune priming can have various levels of specificity (Boraschi et al., 2024; Milutinović and Kurtz, 2016), which was mostly studied at the whole-organism level, ideally using fully factorial priming-challenge approaches with either the same or different pathogens used for priming and challenge (Little and Kraaijeveld, 2004). Such studies have shown that immune priming can be highly specific and able to discriminate between strains of bacteria (Little et al., 2003; Roth et al., 2009), parasite genotypes (Portela et al., 2013), or sibships (Kurtz and Franz, 2003). Immunity leading to resistance at the whole-organism level may consist of intermingled specific and unspecific components (Ng et al., 2024). While unspecific components could be explained by well-described innate immune mechanisms, specific components need further attention. Accordingly, attempts to identify receptors that could enable such fine-scale discrimination led to the description of many, often taxon-specific immune receptor systems that are diversified in the germline, or might even be produced via somatic diversification. An intensively studied example is the alternatively spliced Down Syndrome Adhesion Molecule (Dscam), where diversification occurs at the level of RNA rather than DNA as in the vertebrate adaptive immune system (Ng and Kurtz, 2020; Watson et al., 2005). Dscam may mediate recognition and effector specificity, serving as an opsonin or immune cell receptor (Figure 2). A recent study provides evidence that experimental reduction of Dscam diversity reduces fitness in *Drosophila* when exposed to bacterial pathogens (Dong et al., 2025), but did not directly test whether this is due to reduced specificity of priming. Further examples are SpTransformer gene family (previously Sp185/333; Smith and Lun, 2017) of sea urchin and fibrinogen-related proteins (FREPs) of molluscs (Zhang et al., 2004). Diversity is here generated through a combination of evolutionary processes that increase receptor gene diversity in the germline, such as gene duplication and diversifying selection, with somatic diversification processes. These may include alternative splicing and hypermutation-like processes; however, the details are not yet fully clear (Buckley and Dooley, 2022; Li et al., 2025). The selection pressures originally driving such diversification likely arose in contexts where the interaction between two metazoans would require a higher level of specificity for the host to be able to distinguish ‘self' from 'non-self'. Such scenarios include metazoan parasites that often express a diverse and quickly changing set of antigens (glycoproteins) on their surface (Moné et al., 2010), but also protection against germline parasitism within the species (Cooper, 2018). Additionally, specificity might arise from non-receptor-based processes, as in cases where specificity results from the recognition of specific nucleotide sequences of pathogens, in particular viruses (Mondotte et al., 2018).

However, specificity in immune priming does not necessarily need a very large receptor repertoire or sequence-specific discrimination. Combinations or synergistic action of a limited set of receptors, such as C-type lectins, including heteromultimers enhancing the recognition potential, could explain specificity (Schulenburg et al., 2008). Indeed, this could also underlie the specificity that can be observed in some mammalian innate memory phenomena. Notably, specific immune priming might only be effective against certain pathogens, in the hypothesis that the immune system will not provide receptors for antigenic motifs an organism has not dealt with during evolution. In *T. castaneum*, specific immune priming seems to mostly occur against bacteria that are natural pathogens of this host (Ferro et al., 2019). Virulence factors of a *B. thuringiensis* strain that can infect the beetle elicit the primed state upon oral exposure, whereas non-pathogenic strains of *B. thuringiensis* do not induce such a priming (Baur et al., 2025; Länger et al., 2023). This indicates that in invertebrates, immune priming could sometimes be connected to immunity that is induced by harmful bacteria, but not commensal microbiota (Nagy and Buchon, 2025), in contrast to other examples where commensals mediate priming (Gomes et al., 2021). This may explain why priming in *T. castaneum* is *B. thuringiensis* strain-specific, whereas priming in *A. gambiae* is cross-reactive, such that priming with gut bacteria protects against *Plasmodium* parasites (Figure 2). Since priming appears to be elicited by pathogen effectors (e.g. virulence factors, toxins) in one case, but by bacterial patterns (microbe-associated molecular patterns, MAMPs) in the other, a distinction between effector-triggered immunity (ETI) and pattern-triggered immunity (PTI)—as is well established in plants—would be highly useful, but is not yet applied in the animal field.

Immune priming in plants augments PTI-related defenses (De Kesel et al., 2021; Walters et al., 2013), which protects against a broad range of biotic stresses (Shu et al., 2023). However, recent studies indicate that longer-lasting immune priming in plants displays a degree of specificity and may even lead to increased susceptibility to other types of stress. For instance, priming 2-week-old *Arabidopsis* seedlings with the stress hormone jasmonic acid (JA), which induces short-term resistance against both necrotrophic pathogens and herbivores (Erb and Reymond, 2019; Pieterse et al., 2012), yields long-term induced resistance against the generalist herbivore *Spodoptera littoralis* that is associated with increased susceptibility to the necrotrophic fungus *Plectosphaerella cucumerina* (*Pc*) and the hemi-biotrophic bacterial pathogen *Pseudomonas syringae* pv. *tomato* DC3000 (*Pst*; Wilkinson et al., 2023). This specificity in long-lasting immune priming of plants, along with trade-offs on resistance to other stresses, can be transmitted to the next generation: progeny of *Pst*-infected *Arabidopsis* is more susceptible to necrotrophic fungi, and vice versa (López Sánchez et al., 2021; Luna et al., 2012). Similarly, progeny of salt-treated plants is more susceptible to both biotrophic and necrotrophic pathogens (López Sánchez et al., 2021). Thus, long-lasting priming in plants can be specific against attackers that are resisted by the same innate immune response as the primary (priming-inducing) stress and may be associated with increased susceptibility to stresses that are regulated by other defense pathways.

## Microbiota

The microbiota, that is the complex microbial communities in an environment, including bacteria, fungi, and viruses, plays a vital role in the health and functioning of plants and animals. In plants, the root-associated microbiota has especially been linked to plant-beneficial functions, such as growth-promoting activities by facilitating mineralization uptake of nutrients, as well as protection against pests and diseases through antagonism, nutrient competition, and priming of the plant’s immune responses (Mendes et al., 2011; Pantigoso et al., 2022). In animals, including humans, the microbiota associated with the body is primarily located in the gut and plays crucial roles in digestion, immune function, and overall health. Gut microbiota breaks down complex carbohydrates, produce essential vitamins, induce host immune system development, and regulate host immunity by maintaining a tight balance between beneficial and harmful microorganisms. Disruptions in this balance lead to dysbiosis and can result in a range of diseases. Thus, the microbiota of plants and animals can form a symbiotic relationship with its host by offering improved nutrition and stress resilience.

In plants, long-lasting defense memory can be achieved via an external microbial pathway involving the recruitment and/or selection of health-promoting soil microbes. Stressed plants typically change their root exudation chemistry in response to stress by pests, diseases, and abiotic stresses (Liu et al., 2023; Rolfe et al., 2019). These root-derived chemicals often have allelochemical effects but can also recruit and select for disease-suppressive soil microbes, such as plant growth-promoting rhizobacteria (PGPR), which are tolerant to these chemicals. The altered root microbiota protects plants against pests and diseases via direct and indirect mechanisms, including competition for nutrients, antibiosis, and/or immune priming (induced systemic resistance; ISR). As these microbes establish a self-perpetuating presence long after the initial stress has subsided, they provide plants with a form of external defense memory (Berendsen et al., 2018; Hannula et al., 2021; Yuan et al., 2018). Microbiota-based memory, also known as disease-suppressive plant-soil feedback or soil legacies, relies on a complex interaction between the soil, the host plant, and the soil microbiota and can offer long-term protection to plants and their progeny (Bakker et al., 2018; Spooren et al., 2024).

It has been known since the early 1990s that selected PGPR mediate ISR, which is based on priming of jasmonic acid (JA)- and ethylene-dependent immune mechanisms (Pieterse et al., 2014). Apart from root colonizing PGPR, mutualistic endophytes, such as arbuscular mycorrhizal fungi, have been reported to induce a phenotypically similar ISR response (Cameron et al., 2013). The signaling pathways and systemic signals controlling ISR are complex and likely vary between plant-microbe interactions. Recent evidence suggests that ISR-eliciting rhizobacteria induce a nutrient-deficiency response that results in a systemic priming of defenses. In *Arabidopsis* (Pozo et al., 2008; Zamioudis et al., 2014), ISR-related priming by rhizobacteria is dependent on the R2R3-type transcription factor MYB72, which activates an iron-limitation response in plants. The MYB72-dependent response results in β-glucosidase BGLU42-dependent exudation of the coumarin scopoletin, which is antagonistic to soil-borne pathogens but provides a favorable environment for the priming-inducing rhizobacteria (Stringlis et al., 2018). Exactly how localized root induction of *MYB72* orchestrates systemic priming of JA- and ethylene-dependent defenses remains unknown; however, since overexpression of *BGLU42* mimics ISR (Zamioudis et al., 2014), it is tempting to speculate that a BGLU42-dependent metabolite in *Arabidopsis* acts as the systemic priming signal.

In invertebrates, immune priming can be dependent on the gut microbiota for two reasons. First, certain symbionts may help the host by establishing a primed immune state, which then helps against pathogens. Such symbiont-mediated immune priming (SMIP) has recently been reviewed (Hoang and King, 2022; Prigot-Maurice et al., 2022). Second, the gut microbiota may contribute to immune priming, in the case of infections that breach the gut barrier. This process has been characterized in detail in mosquito *Anopheles gambiae* upon priming with the malaria parasite *Plasmodium* (Rodrigues et al., 2010; Ramirez et al., 2015). *Plasmodium* ookinetes disrupt the peritrophic membrane barrier that normally prevents the gut microbiota from coming into direct contact with gut epithelial cells. By inducing the release of a mosquito lipoxin/lipocalin complex, the released gut microbiota triggers a long-lived response of hemocytes in the mosquito hemocoel, leading to enhanced immunity to bacteria that indirectly reduces the survival of *Plasmodium* parasites upon reinfection. On the other hand, the induction of memory in *An. albimanus* was specific against *P. berghei* and independent of midgut bacteria (Contreras-Garduño et al., 2015). The absence of bacteria by antibiotic treatment and injection of normal flora bacteria from mosquitoes into the hemocoel indicates that responses were specific against the parasite and associated with biphasic antimicrobial peptide (AMP) expression.

Oral immune priming in *T. castaneum* also depends on the presence of the gut microbiota (Futo et al., 2015), and oral priming leads to changes in microbiota composition (Korša et al., 2022; Figure 2). However, in contrast to cross-protection achieved through priming in the mosquito, the reaction shows specificity regarding the bacterial strain used for priming and challenge (Futo et al., 2017), implying the involvement of additional yet unknown mechanisms. Alteration of the gut microbiota in *T. castaneum* led to a decrease of immune priming in populations with experimentally evolved priming response, whereas experimentally evolved constitutive resistance remained unaffected, again demonstrating that priming and resistance might have a different mechanistic basis (Prakash et al., 2023).

Symbiont-mediated immune priming in invertebrates might be achieved by the sustained activation of host immune components, such as AMPs, by the symbiont, which itself has achieved tolerance or resistance. Long evolutionary associations with the symbiont can lead to more specific reciprocal adaptations, such as the production of specific AMPs by the host in specialized cells or tissues such as bacteriocytes (Login et al., 2011). Co-evolution of host and protective symbiont can also lead to strain specificity, that is protective priming is achieved only for certain host and symbiont genotypes, as in the case of *Wolbachia* (Prigot-Maurice et al., 2022). Symbiont-mediated and pathogenic priming may interact or interfere with each other. In *Armadillidium vulgare* isopods, the *Wolbachia* symbiont-mediated priming protects against *Salmonella enterica*, but protection declines with host age, and pathogen-mediated priming becomes visible again in old host individuals (Prigot-Maurice et al., 2021). Cockroaches, like mice, exhibit significant variation in susceptibility to oral infection with *S. typhimurium*, which could be related to immune priming of the host by several low abundance bacterial taxa in the gut microbiota, leading to enhanced AMP production (Turner et al., 2024). In summary, the microbiota seems to be important for immune priming in plants and invertebrates, although the exact mechanisms are often less clear, and its contribution to immune priming specificity is unknown (Contreras-Garduño et al., 2016).

## Evolution of immune priming

All organisms face pathogens and parasites and have evolved a wide range of immune defenses against them. Depending on the frequency and timescale of exposure, either constitutive, innate, or induced, phenotypically plastic responses that remember a previous attack are predicted to evolve (Boots and Best, 2018; Lui, 2000; Mayer et al., 2016). The field of eco-immunology (Schmid-Hempel, 2005; Schulenburg et al., 2009) highlights ecological conditions under which forms of constitutive or induced defenses may evolve, and it puts the costs of these reactions into focus. Constitutive defenses are expected to be more costly, because energy is needed to sustain the immune effectors, and these may also incur immunopathological costs or harm beneficial commensals. Priming-mediated responses, where defense effector mechanisms are not directly upregulated but kept in a more alert state for later challenge, may combine the advantages of enhanced disease protection and low costs (van Hulten et al., 2006). In plants, costs of priming can arise from suppression of innate defenses to other environmental stresses through antagonistic signaling cross-talk (Spoel et al., 2009; Van der Does et al., 2013; Yasuda et al., 2008), which can even affect the progeny of stressed primed plants (López Sánchez et al., 2021; Luna et al., 2012). Nevertheless, it can be assumed that under a large range of conditions, selection pressure exists to evolve induced immune responses or immune priming.

Unsurprisingly, basically all organisms have evolved the ability to develop forms of immune priming. Among animals, invertebrates represent more than 95% of all animal species, while over 400,000 plant species take up 80% of Earth’s biomass across its biomes. It is therefore foreseeable that the mechanisms of immune priming are enormously diverse. Immune priming consists of evolutionarily conserved mechanisms as well as taxon- or even species-specific mechanisms. For example, certain pattern recognition receptors such as Toll-like receptors (TLRs), as well as effectors such as antimicrobial peptides (AMPs), are involved in immune priming responses and predate the evolution of vertebrates (Lazzaro et al., 2020; Leulier and Lemaitre, 2008). Likewise, immune mechanisms involved in priming such as phagocytosis are evolutionarily ancient, and even sponges, the evolutionarily oldest metazoans, possess phagocytic cells (Müller et al., 1999). However, some mechanisms involved in immune priming are more recent evolutionary developments. For example, alternatively spliced Dscam evolved within arthropods and is not found in other invertebrates or vertebrates, which possess only a few isoforms of Dscam (Armitage et al., 2012).

On a microevolutionary scale, a few recent studies have provided evidence that immune priming can evolve rapidly, based on existing genetic diversity among and within populations. For example, Khan et al., 2016 showed that populations of the red flour beetle *T. castaneum* vary in their immune priming responses. Making use of such variation, they were able to experimentally evolve plastic immune priming versus constitutive resistance against the entomopathogen *B. thuringiensis* within 11 generations (Khan et al., 2017). Using the same host organism, Ferro et al., 2019 showed that the specificity of the immune priming reaction can evolve within 14 generations of experimental evolution. Interestingly, increased specificity only evolved against *B. thuringiensis*, but not against other bacteria, showing that the evolutionary potential of immune priming might be limited to naturally encountered pathogens. This evidence suggests that the benefits of immune priming favor its occurrence. However, immune priming is also evolutionarily costly. For example, in insects, immune priming is traded off with metabolic rate, development, and reproduction in terms of mate choice and hatching success (Contreras-Garduño et al., 2019; Contreras-Garduño et al., 2014).

In plants, evidence is emerging for a functional link between the stress-induced epigenetic changes driving long-term immune priming and the evolution of the plant’s innate immunity. While heritable priming disappears in the absence of stress (Stassen et al., 2018), multigenerational exposure to disease stress increases the epi-mutation rates (Zhang et al., 2024a). Interestingly, maximum epigenetic variation between naturally occurring *Arabidopsis* ecotypes occurs at defense-related genes within highly variable TE-rich genomic regions (Kawakatsu et al., 2016), suggesting a functional link between epigenetic and genetic variation in plant immunity. Under stress-free conditions, TEs are epigenetically silenced to prevent mutagenic damage to essential housekeeping genes. Accordingly, widespread DNA hypomethylation of TE-rich regions can lead to transcriptional activation of TEs and increased transposition with potentially mutagenic effects (Hannan Parker et al., 2022). Experimental support for this hypothesis came from multigenerational studies of *Arabidopsis* epigenetic recombinant inbred lines (epiRILs), which are genetically identical but vary in DNA methylation of TE-rich genomic regions (Johannes et al., 2009). Activated TEs within this epiRIL population showed a directional preference for integration in/near environmentally responsive defense genes, including immune regulatory genes, which was driven by histone variant H2A.Z (Quadrana et al., 2019). Furthermore, an epigenomic and bio-climatic survey of >1000 naturally occurring *Arabidopsis* accessions provided evidence for variable TE transposition rates and positive selection of recent TE insertion polymorphisms. This signature was particularly pronounced in accessions at the (more stressful) borders of the species’ ecological niche (Baduel et al., 2021). Since environmental stress is known to induce DNA hypomethylation at TE-rich regions (Hannan Parker et al., 2022), these results indicate that prolonged periods of stress accelerate genetic diversification of immune regulatory genes. Hence, stress-induced DNA demethylation, which drives immune priming within and across plant generations, also acts as a directional catalyst in the evolution of plant immunity to facilitate adaptation to stressful environments.

## Applications of immune priming

Knowledge about immune priming in animal and plants has greatly enhanced our understanding of how immune systems work. Moreover, this knowledge is increasingly translated into beneficial applications, ranging from vector control of human diseases to aquaculture management and the development of novel technologies in crop protection against pests and diseases.

Immune priming holds major potential for applications in crop protection. As outlined in a recent review (Flors et al., 2024), there is increased interest in the exploitation of plant immune priming and induced resistance in crop protection. Current crop protection strategies rely heavily on pesticides and selective breeding for single resistance (R) genes. However, concerns about the environmental impact of pesticides and the erosion of R genes due to co-evolving pests and diseases have led to renewed interest in priming as a way to enhance quantitative resistance in genetically susceptible crop species (Yassin et al., 2021). Although plant immune priming can be associated with costs, such as reduced yield or increased susceptibility to other stresses (López Sánchez et al., 2021; van Hulten et al., 2006; Yassin et al., 2021), when applied in the right context, it can offer commercially attractive levels of protection. Indeed, there is a growing market for biologicals labeled as IR inducers in plants. Examples include ActiGard (Syngenta), LifeGard (CertisUSA); Biotrinsic (IndigoAg), Regalia (Marrone Bio Innovations), and Vacciplant (Helena). Besides being effective against taxonomically unrelated pests and diseases, priming can help reduce pathogen loads to protect single R genes against co-evolving pests and diseases. Recent insights into the (epi)genetic control of root exudates that recruit and/or select for priming-inducing soil microbes (Cawood and Ton, 2025), as well as the latest knowledge about the molecular perception and downstream signaling of priming by chemically induced resistance agents, provide tangible breeding targets for selecting a new generation of crop varieties that are better able to recruit priming-inducing soil microbes and respond to chemical priming agents. Perhaps the most exciting translational opportunities arise from emerging knowledge about epigenetic mechanisms driving heritable priming in plants. Proof-of-concept studies with epigenetic recombinant inbred lines (epiRILs) of *Arabidopsis* have demonstrated that introgression of reduced DNA methylation in TE-rich pericentromeric regions provides high levels of heritable disease resistance without growth costs (Furci et al., 2019). However, due to the much higher number of TEs in crop genomes, translating this technology to crops requires more adjustable approaches to induce sufficient DNA demethylation for heritable immune priming, while simultaneously preventing the widespread activation of TEs that could lead to lethality and sterility (Cooper and Ton, 2022). Exploiting transgenic approaches that enable spatio-temporal ectopic control of DNA demethylase activity, as well as recent advances in the use of CRISPR-dCas constructs for epigenomic editing (Harris et al., 2023), offers technological opportunities to achieve this goal.

Knowledge of immune priming in insects will be instrumental in understanding how insect pests in agriculture achieve resistance against biological control measures. For instance, immune priming has been demonstrated against many biological control agents, such as *Bacillus thuringiensis*, *Metarhizium anisopliae*, and parasitoid wasps (Medina Gomez et al., 2018; Roth et al., 2009; Trauer-Kizilelma and Hilker, 2015). While priming may help insect pests survive these control agents, it is currently unclear whether the immediate survival benefits provided by such phenotypically plastic immunity facilitate or hinder the evolution of resistance in the long term (Pfennig, 2021).

Invertebrates, particularly insects, are important vectors of human diseases. Priming the immune system of such vectors may reduce their transmission potential and thus provide an alternative to the usually difficult attempts to eradicate vectors. Dengue, Zika, Chikungunya, and malaria are prominent examples of such vector-borne diseases (VBD), which pose significant public health problems and require novel approaches to interrupt their transmission. Mosquitoes *An. albimanus* primed against *Plasmodium* prevent parasite development in the midgut (Contreras-Garduño et al., 2015). A similar result has been obtained in mosquitoes *Ae. aegypti* primed with dengue virus (DV), where protection is reached after the oral feed with inactive viruses (Vargas et al., 2020). One setback is the practical application of this strategy. Oral feed is not convenient for field application. However, in *Ae. aegypti*, protection and memory can be induced from the larval stages (aquatic instars), making the adults resistant to infection (Vargas et al., 2020). This procedure can make the priming effect more feasible and practical. It is fundamental to investigate if the priming effect can be reached from the ontogeny (larval stages) in different VBD vectors, facilitating the priming application in the field. One attractive priming characteristic is its transgenerationality, which would allow primed larvae or adults to disseminate resistance through their descendants (Cime-Castillo et al., 2023). This last approach deserves a deeper investigation and analysis to determine its limits and potential application for mosquitoes, since transgenerationality may substantially depend on the persistence of the priming agent in the insect environment.

Since pollinators are economically relevant, there is a strong interest in innate immune memory of honeybees against diseases. ‘Vaccination’ strategies for honeybees, particularly aimed at preventing American foulbrood (AFB), caused by the bacterium *Paenibacillus larvae*, have gained attention in recent years (Dickel et al., 2022). The development of effective vaccination methods is crucial due to the severe impact of AFB on honeybee populations and the subsequent economic consequences for beekeepers. A promising approach is to exploit TGIP, the trans-generational immune priming that allows transmitting resistance to the progeny, as a potential vaccination strategy against AFB (Dickel et al., 2022). Indeed, when honeybee queens are exposed to heat-killed *P. larvae*, their offspring exhibit enhanced immune responses without apparent significant trade-offs in colony performance (Leponiemi et al., 2023). An AFB vaccine targeting honeybee queens has been conditionally approved by the United States Department of Agriculture FDA in 2023, and AFB-vaccinated honeybee queens are already commercially available.

Another field where immune priming has large potential for application is aquaculture (Montagnani et al., 2024; Wang et al., 2025). Invertebrates account for one third of global aquaculture production worldwide, encompassing species of diverse phylogenetic groups, mostly crustaceans (e.g. shrimp, crawfish, crab) and molluscs (‘shellfish’, e.g. oysters, scallops, mussels), but also echinoderms (sea cucumber). Invertebrate aquaculture is in rapid growth but suffers from economic risks due to the occurrence of infectious diseases caused by viruses (e.g. white spot syndrome virus [WSSV] in shrimp), bacteria (e.g. vibriosis, affecting a wide range of crustaceans and shellfish), fungi (e.g. Fusarium species in shrimp), and protozoan parasites (e.g. Perkinsosis in oysters). Thus, the field of aquaculture shows a strong interest in immune priming. First vaccination trials for shrimp have been developed already in the 1990s (Amatul-Samahah et al., 2020). While these treatments are not directly comparable with conventional vaccines in vertebrates, they can provide protection, either as rather unspecific immunostimulants (such as formalin-killed *Vibrio*) that reduce infections due to the induction of a strong immune activation, or as more specific treatments targeting certain viruses using RNA interference. The economic interest in aquaculture has also driven basic research in immune priming, in the attempt to unravel the large repertoire of humoral and cellular immune mechanisms underlying the improved disease resistance of aquaculture animals treated with a variety of ‘priming inducers’ (Wang et al., 2025).

A steeply rising field where such knowledge could be applied is the farming of insects as feed and food (Ali Mohammadie Kojour et al., 2022; Grau et al., 2017; Madau et al., 2020). Some insects used for mass production, such as black soldier fly larvae, crickets, and mealworms, are closely related to the animal models in basic research on immune priming, which may facilitate developing applied strategies for disease prevention.

Finally, application of immune priming may help against species extinction. Research on the sea anemone *Metridium senile* has provided insights into the molecular basis of immune priming in cnidarians, which include corals. Brown and Rodriguez-Lanetty, 2015 have shown that sea anemones that had previously encountered a pathogen under sub-lethal conditions had an up to sevenfold higher survival than naïve anemones during a subsequent lethal challenge 2 and 4 weeks later. However, anemones challenged 6 weeks after the sub-lethal exposure showed no increased survival. They suggest that this priming of the defense response could be ecologically relevant if pathogen encounters are restricted to short seasons characterized by high stress (Brown and Rodriguez-Lanetty, 2015).

However, there are also risks that need to be considered when applying immune priming. We currently lack knowledge of how pathogens evolve when confronted with innate memory responses. If immune priming is less effective than the highly specific adaptive immune memory, it may still enable pathogen transmission and thus has been suggested to bear the risk that pathogens evolve increased virulence (Gandon et al., 2001). Indeed, using an experimental evolution approach, Hoang et al., 2024 recently found that symbiont-mediated priming by a natural microbiota member, *Pseudomonas berkeleyensis*, in *C. elegans* led to increased virulence of the pathogen *P. aeruginosa*. Experimental evolution of *B. thuringiensis* in immune-primed *T. castaneum* hosts gave rise to increased variation in virulence among replicate lines, which may favor adaptation (Korša et al., 2025). This underscores the need to consider pathogen evolution in response to innate immune memory when thinking about applications of immune priming.

Thus, while applications of immune priming bear large potential in diverse fields, further studies on its microevolutionary consequences are required. The diversity of mechanisms involved across different species and even within a species poses additional challenges to the transfer of knowledge to different diseases and further species. Moreover, it needs to be considered that immune priming bears costs in terms of energy demands and potential self-harm of activated immunity (Sadd and Siva-Jothy, 2006). We will not be able to fully exploit the potential of immune priming applications without a more complete understanding of its embedding into the cellular and organismal metabolism, and its potential costs and constraints (Armitage et al., 2003; Méndez-López et al., 2024; Schmid-Hempel, 2005).

## Conclusions and perspectives

For the present article, researchers of plant and animal immune systems have teamed up to improve understanding of forms of immune memory in innate immune systems. Such phenomena exist in plants and most, potentially all, invertebrate animal taxa, as well as in the innate immune system of vertebrates. Related phenomena of priming and memory of stress responses have even been described in bacteria and fungi, allowing for comparisons with priming in plants (Hilker et al., 2016). A recent review has compared plant SAR with vertebrate trained immunity, concluding that many cross-kingdom similarities provide potential for both agricultural and medical practices (Conrath, 2025). In the present work, we examined immune priming in plants and invertebrates, as well as trained immunity in vertebrates. These phenomena display broad commonalities at the phenomenological level, while their underlying mechanisms usually differ. Our overview focusing on the aspects of epigenetics, signaling, specificity, microbiota, evolution, and upcoming applications gives rise to a number of conclusions and perspectives for future work.

### An evolutionary viewpoint on concepts

Comparative immunology (Boehm, 2025; Cooper, 2018) helps identify which of the highly diverse processes involved in innate immune memory are based on evolutionary homology, which ones have evolved in parallel in different taxa, and which are taxon-specific innovations. The commonalities, on the other hand, provide a basis for unifying conceptual approaches. Without clear concepts, research in the field of innate immune memory bears the risk to advance in different ways. However, we should acknowledge that one single concept of innate immune memory might be out of reach, that is we need to accept conceptual pluralism that stimulates the discussion. We thus suggested different types of concepts for ‘immune priming’, defined broadly in a phenomenological way, vs. ‘trained immunity’ defined more narrowly by including the involved mechanisms identified in vertebrates. This allows analyzing whether ‘trained immunity’ also exists in plants and invertebrates, rather than simply adopting this term for all immune priming. Currently none of the described examples of immune priming in plants and invertebrates fulfils all defining criteria of trained immunity, while similarities exist regarding the involvement of epigenetic processes or metabolic reprogramming. However, sharing possibly evolutionary ancient components contributing to a trait does not necessarily mean that the trait as such is based on evolutionary homology. Moreover, we should avoid generalizations that have hampered progress in the early days of invertebrate immunology, where the absence of the mechanisms responsible for adaptive immunity in vertebrates was used as an argument against the existence of invertebrate immune memory (Cooper et al., 1992; Klein, 1989; Klein, 1997). An approach driven by curiosity leading to phenomenological observations followed by the search for novel mechanisms has been fruitful and has become even more promising nowadays, where genomic information can be obtained even from non-model organisms. We should continue using the rich source of the millions of species of plants and animals with their unique environmental adaptations driving evolutionary innovations in their immune systems. Yet unknown phenomena and mechanisms of innate immune memory await discovery and may teach us further general principles of immunity.

### Priming elicitors

Immune priming in plants and invertebrates is not a universal response to all non-self patterns but instead occurs selectively in response to particular cues. In many hosts, naturally occurring pathogens seem more likely inducers of priming. Accordingly, priming specificity seems limited to relevant risks, unable to discriminate between all potential antigens. Virulence factors of pathogens could be targets as priming elicitors. However, if a parasite or pathogen causes severe damage, the immune response might be hyperactivated, and host immediate survival might be more relevant than memory. The type of priming stimulus, that is biotic (e.g. an infectious agent) or abiotic/environmental (e.g. temperature shock, mechanical stress) should also be considered. Protection can be reached in both cases, but specificity, transgenerationality, and mechanisms can differ.

### Local and systemic priming

Priming can be restricted to certain cell types or organs, but it can also affect the whole organism, becoming systemic. Both plants and animals use signaling molecules to transmit the primed state to distant cells, although the identity of these molecules differs — for example, salicylic acid (SA) in plants versus cytokines in animals, where mobile immune cells further facilitate systemic responses. In vertebrates, most studies on trained immunity have focused on specific immune cell populations that acquire a trained state, with only a few examples demonstrating how this response can become systemic and persist beyond the relatively short lifespan of innate immune cells (Mitroulis et al., 2018). In invertebrates, the route of infection may determine which priming reactions occur—for instance, gut-based responses following oral priming (Baur et al., 2025). Initially, local responses can also spread and result in systemic priming (Deng et al., 2025; Gomes et al., 2021). However, little is currently known about potential organ-specific priming in invertebrates (Marino et al., 2023), its maintenance through metamorphosis — particularly in holometabolous insects — or its transmission across generations. Similarly, in plants, little is known about organ- or tissue-specific priming responses, although it is evident that roots and leaves differ in their immune responsiveness to non-self and damaged-self elicitors (Zhou et al., 2020). Across invertebrates and plants, it appears that epigenetic modifications are ultimately required for long-lasting systemic priming (Conrath, 2025; Gomes et al., 2022; Ziogas et al., 2025). To better understand the fundamental principles of systemic priming in animals, research could benefit from further cross-kingdom comparisons with priming in plants (Conrath, 2025).

### Costs of priming

Costs of immunity can be broadly divided into constitutive costs needed to maintain an immune system even in the absence of an infection (e.g. the energy and resources invested into keeping immune cells ready) and defense costs (e.g. the energy and resources needed during an immune reaction). Immune priming is a way to reduce constitutive costs; nevertheless, energy and resources are needed to keep the system alert. Immune priming is therefore closely tied to energy availability and metabolic processes. This energy is typically diverted from other physiological processes, such as growth, reproduction, or maintenance. Upon immune challenge, organisms undergo metabolic shifts to support the immune response. Epigenetic processes involved in priming may themselves be energetically demanding, thus the link between epigenetics and metabolism seen in vertebrate trained immunity and occasionally in immune priming appears meaningful. Nutrient availability affects invertebrate immune response and memory. For example, dietary carbohydrates and lipids influence immune gene expression and memory formation. However, several aspects require further investigation, including the specific metabolic pathways involved in immune priming, the role of hormones and neurotransmitters in regulating energy metabolism and immune response, and the impact of environmental factors (e.g. temperature, nutrition) on priming. Such knowledge is relevant also for application of immune priming, as it, for example, depends on the specific conditions whether or not it is economic to make routine use of priming inducers in farmed animals in aquaculture or insect mass production. In addition, a better understanding of the costs of immune priming in plants could enable more effective and reliable applications in crop protection—for example, through the development of formulations that combine low concentrations of priming agents with minimal side effects on yield (Yassin et al., 2021), and through the use of epigenetic breeding technologies to introduce stable heritable priming traits into the germplasm of crop varieties (Cooper and Ton, 2022).

### Research implications

Understanding invertebrate and plant immune memory can provide novel strategies for developing vaccines and other treatments for economically and ecologically relevant species. There are several examples, such as in bees, shrimp, and mosquitoes, where immunization can protect against pathogens and their transmission. Moreover, this approach will allow analyzing the conservation of immune mechanisms between plants, invertebrates, and humans, providing clues for developing new treatments for human diseases. It is worth mentioning that vertebrate vaccines were applied before their molecular mechanisms were understood, based on their protective efficacy. Finally, immune priming bears a currently under-appreciated relevance in the context of the ‘One Health’ approach (Brown et al., 2024; Lerner and Berg, 2015). This integrated and interdisciplinary approach aims to understand and address the interconnected health of humans, animals, plants, and the environment. Immune priming helps organisms to protect themselves against disease threats that often increase due to perturbed environmental conditions. Immune priming could prevent diseases in domestic and wildlife animals. Monitoring immune parameters that are indicative of priming in animals may even serve as sentinels for the early detection of zoonotic diseases.
